# A New Dressing

**Published:** 1897-12

**Authors:** 


### A New Dressing.

Dr. Reed: I have here a form of dressing, new in my experience, to which my attention was called in Mexico. It is nothing more nor less than asbestos put through a process of refinement. It has several virtues. In the first place, it is very soft. In the next place, it is capable of complete sterilization. Place some of this dry on a plate, put a few drops of alcohol on it and ignite it and the sterilization is complete. The absorbent properties are better than those of cotton. When dampened it becomes very smooth, absolutely non-irritating, and if then used as a sponge you will not disturb surface lymph deposits or granulations. Enclosed in a little gauze, it makes a perfect sponge, as sensitive as the most refined of sponges. It can be had in various forms. For instance, it is gotten up in the form of a wick. Here is a paper perforated, used for various dressings. It is thoroughly sterilizable. And here is a wick for intra-uterine packing, which has been reported very satisfactory, but I would hesitate to use it, because it seems to me you could not pull it out without it breaking.

